# Brain Ischemia Significantly Alters microRNA Expression in Human Peripheral Blood Natural Killer Cells

**DOI:** 10.3389/fimmu.2020.00759

**Published:** 2020-05-14

**Authors:** Ying Kong, Shiyao Li, Xiaojing Cheng, Honglei Ren, Bohao Zhang, Hongshan Ma, Minshu Li, Xiao-An Zhang

**Affiliations:** ^1^Department of Neurology, Tianjin Neurological Institute, Tianjin Medical University General Hospital, Tianjin, China; ^2^Tianjin Neurological Institute, Key Laboratory of Post-neurotrauma Neuro-Repair and Regeneration in Central Nervous System, Ministry of Education and Tianjin City, Tianjin, China; ^3^Department of Anesthesiology and Critical Care Medicine, Johns Hopkins School of Medicine, Baltimore, MD, United States; ^4^Department of Imaging, The Third Affiliated Hospital of Zhengzhou University, Zhengzhou, China; ^5^Center for Neurological Diseases, The Third People's Hospital of Datong, Datong, China

**Keywords:** stroke, natural killer cells, immunosuppression, molecular regulation, microRNA

## Abstract

Brain ischemia induces systemic immunosuppression and increases a host's susceptibility to infection. MicroRNAs (miRNAs) are molecular switches in immune cells, but the alterations of miRNAs in human immune cells in response to brain ischemia and their impact on immune defense remain elusive. Natural killer (NK) cells are critical for early host defenses against pathogens. In this study, we identified reduced counts, cytokine production, and cytotoxicity in human peripheral blood NK cells obtained from patients with acute ischemic stroke. The extent of NK cell loss of number and activity was associated with infarct volume. MicroRNA sequencing analysis revealed that brain ischemia significantly altered miRNA expression profiles in circulating NK cells, in which miRNA-451a and miRNA-122-5p were dramatically upregulated. Importantly, inhibition of miR-451a or miR-122-5p augmented the expression of activation-associated receptors in NK cells. These results provide the first evidence that brain ischemia alters miRNA signatures in human NK cells.

## Introduction

Infectious complications such as pneumonia and urinary tract infection account for about 20% of deaths in stroke victims. Evidence indicates that stroke induces severe suppression of systemic immunity, rendering patients susceptible for post-stroke infections (1–3). A hallmark of stroke-induced immunodepression is lymphopenia, which is defined as a significant reduction of peripheral blood lymphocyte count (4–6). Accumulating evidence has revealed that stroke-induced brain injury activates neurogenic pathways including sympathetic innervation and hypothalamic-pituitary-adrenal (HPA) axis, resulting in suppression of systemic immunity (4, 5, 7). However, the molecular mechanisms through which stroke suppresses systemic immune response in peripheral blood remain poorly understood.

As non-coding RNAs with a length of 18-25 nucleotides, microRNAs (miRNAs) are recognized as major molecular switches that act as post-transcriptional repressors of protein-coding target messenger RNAs (mRNAs). By binding to the 3′-untranslated region (3′UTR), miRNAs induce the decay of mRNA molecules of target genes and inhibit their expression (8). The critical role of miRNAs in directing systemic immune responses has become increasingly clear. In particular, specific miRNAs are being identified in regulation of the development, function, and survival of peripheral immune cells in the setting of infections (9, 10). In human stroke, the alterations of miRNA expression pattern in whole blood cells have been reported (11, 12), suggesting a profound impact of stroke on peripheral immune system. However, the alterations of miRNAs in specific human immune cell subsets after stroke and their potential impact on host immune defense remain unknown.

Natural killer (NK) cells are cytotoxic innate lymphocytes that are critical for early host defenses against pathogens (13). In this study, we characterized the immune competence of human peripheral blood NK cells after acute ischemic stroke and performed unbiased miRNA sequencing analysis of NK cells after brain ischemia. We identified specific miRNAs such as miR-451a and miR-122-5p that were significantly upregulated in human NK cells after acute ischemic stroke. Of interest, we found that inhibition of miR-451a or miR-122-5p augmented the expression of activation-associated receptors in human peripheral blood NK cells after acute ischemic stroke.

## Materials and Methods

### Study Population

A total of 28 stroke patients were recruited from Tianjin Medical University General Hospital. A number of inclusion criteria was adopted: (i) >18 year of age; (ii) acute onset of focal neurological deficit consistent with acute ischemic stroke; (iii) anterior-circulation ischemic stroke defined by magnetic resonance angiography (MRA) or MRI; (iv) onset of symptom to admission within 24 h after stroke. Exclusion criteria were patients with: (i) hemorrhagic stroke; (ii) evidence of other diseases of the central nervous system; (iii) preexisting neurologic disability (a score >2 on the mRS); (iv) patients with any history of bradyarrhythmia or atrioventricular blocks; (v) concomitant use with antineoplastic, immunosuppressive, or immune-modulating therapies; and (vi) macular edema. Eighteen age-and sex-matched control subjects were recruited into this study. The demographic and clinical features of all the patients and control subjects are summarized in Supplemental Table 1. The present study protocol and supporting documentation were approved by the Ethics Committee of Tianjin Medical University General Hospital.

### Flow Cytometry

Peripheral blood samples were collected from control or ischemic stroke subjects with ischemic stroke within 3 days (acute stage) and 7–10 days (subacute stage) after stroke onset. Thereafter, mononuclear cells were isolated from the whole-blood specimens and immediately stained with fluorescent-labeled antibodies. In the case of IFN-γ staining, 2 × 10^6^ PBMC were cultured in the cell medium (RPMI 1640 + 10% heat-inactivated fetal bovine serum + 1% penicillin-streptomycin + 1 mM L-glutamine) for 4 h at 37°C in a CO_2_ incubator in the presence of ionomycin (1 μM; Merck), phorbol-myristate-acetate (PMA, 10 ng/mL; Sigma), and GolgiPlug containing brefeldin A (1 μg/mL; BD biosciences) before flow cytometry analysis. For the staining of the intracellular molecules, cells were fixed and permeabilized using commercial kit (BD Pharmingen) according to the manual. All antibodies were ordered from Biolegend (San Diego, CA, USA), unless otherwise indicated. Antibodies used in this study included CD3 (UCHT1), CD56 (HCD56), CD69 (FN50), NKG2D (1D11), CD27 (O323), CD158 (HP-MA4), IFN-γ (4S.B3), and Perforin (dG9). Flow cytometry was performed using a FACS Aria III (BD Bioscience, San Jose, CA, USA), and data were analyzed by Flow Jo version V10 (flowjo.com).

### Cell Sorting and RNA Extraction

For the miRNA sequencing analysis, human peripheral blood mononuclear cells (PBMCs) were isolated from blood samples of two individual patients with ischemic stroke and two individual healthy controls via Ficoll density gradient centrifugation (GE Healthcare Bio-Sciences, USA), respectively. After staining with anti-CD3 and anti-CD56 antibodies, NK cells (CD3^−^ CD56^+^) were sorted by a FACSAria cell sorter (BD Biosciences, San Jose, CA, USA). The purity of the recovered NK cells was typically >98%. Total RNA was extracted from purified NK cells using TRIzol reagent (Invitrogen, Grand Island, USA) according to the manufacturer's standard protocol. The concentration of RNA was determined with a NanoDrop ND-1000 spectrophotometer (NanoDrop Technologies, Wilmington, DE, USA), and quality assessment was made by an Agilent 2100 Bioanalyzer (Agilent, Waldbronn, Germany). The OD260/280 ratio of all of the samples was within a range of 1.8–2.2. 28S/18S rRNA ratio ≥1.8, and an RNA integrity number ≥8.

### miRNA Sequencing

Total RNA was subjected to multiplexed small RNA cDNA library preparation. Library preparation entails ligation of barcoded 3′ adapters, pooling of samples, ligation of a 5′ adapter, reverse transcription, and polymerase chain reaction (PCR), as previously described (14). Libraries were used Agilent 2100 Bioanalyzer and ABI StepOnePlus Real-Time PCR System checking the quality and yield. Then, libraries were sequenced on an Illumina HiSeq sequencer, and the information obtained was analyzed by an automated computer pipeline to decode and annotate small RNA reads. Normalization of miRNA reads was performed by dividing each miRNA read frequency by the total number of miRNA sequence reads within the subsample, thereby correcting the variable sequencing depth in each subsample.

### Bioinformatics Analysis

The DESeq algorithm was used to test the differential expression of miRNA among the groups. The differentially expressed miRNAs were chosen as follows: fold change >2 or <0.5; *p* < 0.05. Target genes of miRNAs were predicted using miRanda and Targetscan. The KEGG database is a resource for understanding high level functions and effects of biological systems (https://www.genome.jp/kegg/). *P*-values were defined by Fisher's exact test, and FDR was calculated by the BH test. Differentially expressed genes were considered to be significantly enriched for KEGG terms with *p* < 0.05.

### Real-Time PCR Quantification of miRNA

Total RNA was extracted with TRIzol Reagent as above described. cDNA generation and real-time PCR were performed using Hairpin-it microRNA qPCR Quantitation Kit (GenePharma, Shanghai, China). All PCR reactions were performed using standard PCR conditions. RNU6 was used as endogenous control. Data were generated using CFX Manager software (Bio-Rad, CA, USA). The results were analyzed based on the 2^−ΔΔ*ct*^ method.

### Cell Isolation and Transfection *in vitro*

NK cells from circulating blood of patients with ischemic stroke and control subjects were isolated by microbeads (Miltenyi Biotec Inc., San Diego, CA, USA) according to the manufacturer's instructions. For transfection, the purified NK cells were divided into 24-well plates containing 500 μL of cultured medium at a density of 2 × 10^5^ cells/well. The transfection reagent siRNA-Mate was purchased from GenePharma (Shanghai, China). In brief, 20 pmol miRNA-451a (5′-AACUCAGUAAUGGUAACGGUUU-3′) and miRNA-122-5p (5′-CAAACACCAUUGUCACACUCCA-3′) inhibitors were used to block miRNA-451a and miRNA-122-5p function, respectively. Negative control miRNA (NC) was transfected parallelly. In brief, 20 pmol hsa-miR-451a inhibitor, hsa-miR-122-5p inhibitor or NC and 2 μl siRNA-mate were diluted with 100 μl opti-MEM. After incubation for 10 min at room temperature, the transfection complex was added to 24-well plates cultured with NK cells. After transfection, NK cells were cultured for an additional 48 h at 37°C in a CO_2_ incubator, and then cells were collected for following experiments.

### Statistics

Graphpad Prism 7.0 software was used for the statistical analysis. Values in the study are presented as means ± SEM. The statistical significance between two groups was analyzed by two-tailed, unpaired Student *t*-test. For analysis of more than two groups, a one-way analysis of variance (ANOVA) was used. The correlation between two variables was analyzed using the Pearson's correlation test. *P* < 0.05 are considered significant.

## Results

### Immune Phenotyping of Peripheral Blood NK Cells in Patients With Acute Ischemic Stroke

**Figure 1:**
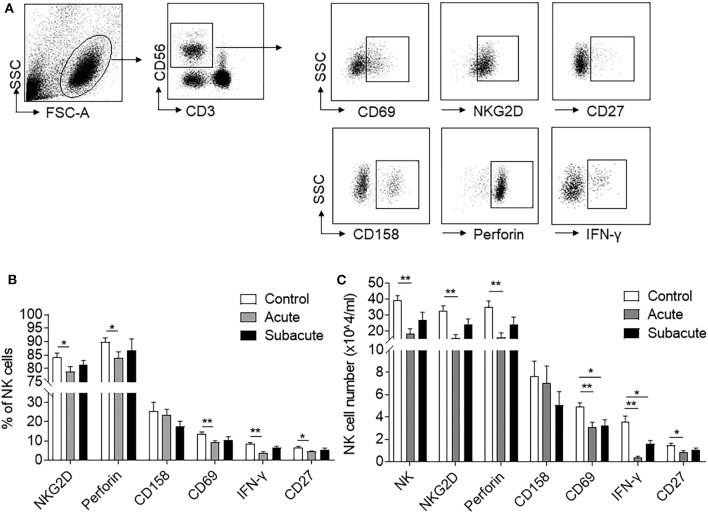
Reduced NK cell number and activity after ischemic stroke. Peripheral blood was obtained from healthy controls and patients with ischemic stroke during acute (<3 days) and subacute (days 7–10) stages. **(A)** Flow cytometry plots show the expression of activation markers (CD69), cytotoxicity receptors (NKG2D and CD158), maturation marker (CD27), and functional proteins (Perforin and IFN-γ) in peripheral NK cells. **(B)** Bar graph shows the cell percent of NK cells that express CD69^+^, NKG2D^+^, CD27^+^, Perforin^+^, or IFN-γ^+^ in healthy control and patients with ischemic stroke during acute and subacute phases. **(C)** Bar graph shows cell number of peripheral NK cells and NK cells that express CD69^+^, NKG2D^+^, CD27^+^, CD158^+^, Perforin^+^, or IFN-γ^+^ in healthy control and patients with ischemic stroke during acute and subacute phases. *n* = 18 for control subjects. *n* = 20 for stroke patients. *p < 0.05 and **p < 0.01. Data are presented as means ± SEM.

To measure the impact of brain ischemia on NK cell activity and immune competence, we used flow cytometry to measure the expression of activation marker (CD69), maturation marker (CD27), functional receptors (NKG2D, CD158), and cytokines (Perforin, IFN-γ) in peripheral blood NK cells from human subjects with acute ischemic stroke and controls subjects. The characteristics of human subjects was shown in Supplemental Table 1. We found that the number of circulating NK cells was significantly reduced after acute ischemic stroke (<72 h after onset) as compared to control subjects, and the loss of NK cells was restored at later time points (days 7–10) in this cohort of patients (control: 39.1 ± 3.2 vs. acute: 18.3 ± 3.1 vs. subacute: 27.2 ± 4.6, × 10^4^/mL, *p* < 0.01; Figures 1A,B). In addition, the percentage and counts of NK cells that express CD69, Perforin, IFN-γ, NKG2D, and CD27 were also reduced during acute stage, but recovered at later time points (Figures 1B,C). These results suggested that brain ischemia significantly but transiently suppresses peripheral NK cell number and activity.

### Brain Infarct Volume as a Major Determinant of NK Cell Suppression in Peripheral Blood

**Figure 2:**
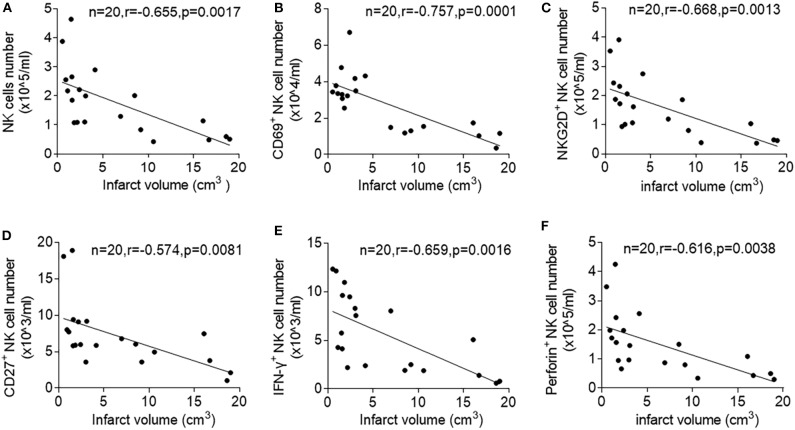
Correlation between infarct volume and circulating NK cell number and activity after ischemic stroke. Peripheral blood was obtained from patients with acute ischemic stroke during the acute stage (<3 days). Linear correlation between stroke volumes and numbers of peripheral NK cells **(A)**, CD69^+^ NK cells **(B)**, (NKG2D ^+^NK cells **(C)**, CD27^+^ NK cells **(D)**, IFN-γ^+^ NK cells **(E)**, and Perforin^+^ NK cells **(F)** in patients with ischemic stroke during acute phase of stroke. Data are presented as means ± SEM.

To determine stroke-specific factors that predict NK cell suppression, we measured the correlation between brain infarct volumes and loss of NK cell number and activity after acute ischemic stroke. Of note, we found that stroke patients with larger infarct volume have more severe loss of NK cell number (*r* = −0.655, *p* = 0.0017) and expression of CD69 (*r* = −0.757, *p* = 0.0001), NKG2D (*r* = −0.668, *p* = 0.0013), IFN-γ (*r* = −0.659, *p* = 0.0016), CD27 (*r* = −0.574, *p* = 0.0081), and Perforin (*r* = −0.616, *p* = 0.038) after stroke onset (Figures 2A–F). These results suggest that the severity of ischemic brain injury determine the extent of NK cell suppression in the periphery.

### Altered Expression of miRNAs in Peripheral Blood NK Cells After Brain Ischemia

**Figure 3:**
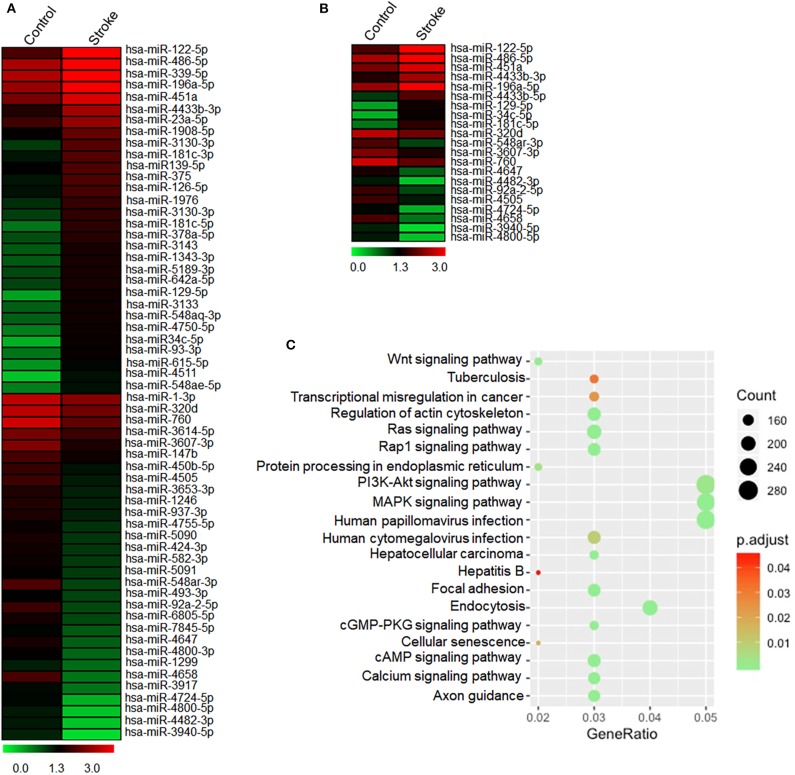
Expressed profile of altered miRNAs in human peripheral blood NK cells after acute ischemic stroke. Peripheral blood was obtained from healthy controls and patients with acute ischemic stroke. NK cells were sorted by flow cytometry. **(A)** The heatmap shows total miRNA alteration (fold change >2) in peripheral NK cells after stroke as compared to control. Red indicates higher expression, and green indicates lower expression. **(B)** The heatmap shows 21 significantly different miRNAs in peripheral blood NK cells after stroke as compared to controls. Red indicates higher expression, and green indicates lower expression. **(C)** KEGG pathway analysis on the targeted genes of 21 differentially expressed microRNAs. The abscissa Gene Ratio represents the proportion of genes of interest in the pathway, and the ordinate represents each pathway. The size of the dots represents the number of genes annotated in the pathway, and the color of the dots represents the corrected *p*-value of the hypergeometric test.

**Figure 4:**
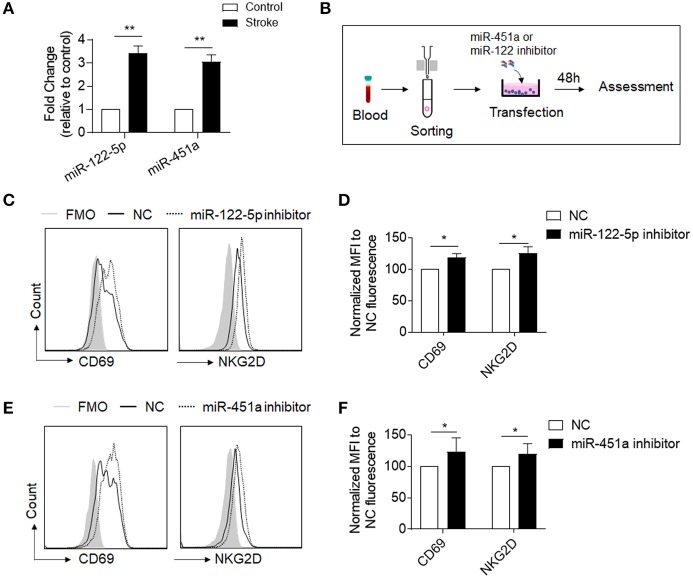
Inhibition of miR-122-5p or miR-451a increases expression of CD69-or NKG2D in NK cells of stroke patients. **(A)** RT-PCR analysis confirmed the miR-122-5p and miR-451a expressions in NK cells of stroke patients and controls. *n* = 8 per group. *p < 0.05. **(B)** Flow chart depicts the experiments we did to evaluate the function of miR-122-5p and miR-451a in purified NK cells of stroke patients. Peripheral blood was obtained from patients with ischemic stroke. NK cells were isolated from the PBMCs by using magnetic beads. After miR-451a or miR-122-5p inhibitor transfection, cells were cultured for an additional 48 h and collected for flow cytometry analysis. **(C,D)** Comparison of CD69-or NKG2D conjunction fluorescence MFI value in NK cells transfected with miRNA-122-5p inhibitor to that with negative control (NC). FMO, Fluorescence Minus One. Bar graphs shows the MFI normalized to NC fluorescence. *n* = 6 per group, *p < 0.05. **(E,F)** Comparison of CD69-or NKG2D conjunction fluorescence MFI value in NK cells with CD451a inhibitor to negative control (NC). Bar graphs shows the MFI normalized to that with negative control (NC). *n* = 6 per group, the data were from three independent experiments. *p < 0.05, **p < 0.01 data are presented as means ± SEM.

To determine the response of NK cell miRNAs to acute ischemic stroke, we performed miRNA sequencing of peripheral blood NK cells obtained from patients with ischemic stroke, followed by unbiased cluster analysis. We identified 117 miRNAs that have more than two-fold changes (either upregulation or downregulation, FPKM >25) in NK cells from stroke patients vs. control subjects. Figure 3A shows the heatmap of the top 60 of the altered miRNAs with a fold change ≥2 and an uncorrected *p* < 0.05. After FDR correction, a total of 21 miRNAs were significantly different in their expression between the control subjects and ischemic stroke patients as shown in Figure 3B. To verify these changes detected by miRNA sequencing, we performed RT-PCR to measure the expression levels of six selected miRNAs (miR-486-5p, miR-23a-5p, miR-92a-2-5p, miR-4647, miR-122-5p, and miR-451a) that are dramatically altered in NK cells after brain ischemia. Consistent with the data of miRNA sequence, the expression of miR-122-5p, miR-451a, miR-486-5p, and miR-23a-5p in peripheral NK cells from the stroke patients were significantly increased, while miR-92a-2-5p and miR-4647 decreased as compared to controls (Supplemental Figure 1 and Figure 4A).

To evaluate the potential functions of 21 differentially expressed miRNAs after stroke, KEGG pathway enrichment analyses were conducted. The top 20 results of the KEGG analysis are presented in Figure 3C. We found that pathways related to infection, signaling pathways involved in the regulation of immune system including MAPK pathway and PI3K signaling pathway, cAMP signaling pathway, as well as cell senescence were list in the KEGG pathway, suggesting that miRNAs may regulate these pathways to influence NK cell activity after acute ischemic stroke.

### Inhibition of miR-451a or miR-122-5p Can Partly Reverse NK Cell Immunosuppression After Stroke *in vitro*

MiRNA-451a and miRNA-122-5p are among the most dramatically upregulated miRNAs in the miRNA-sequence data, as shown in Figure 3B, consistent with RT-PCR data (Figure 4A). Considering their prominent role in suppression of cell functions through PI3K and cAMP signaling pathway (15–18), we therefore examined the potential contribution of miRNA-451a and miRNA-122-5p to the suppression of circulating NK cells after brain ischemia. For this purpose, we transduced commercial inhibitors of miRNA-451a or miRNA-122-5p into NK cells that were isolated from peripheral blood of patients with acute ischemic stroke (Figure 4B). We found that a significantly increased expression of activation-associated surface makers CD69 and NKG2D in NK cells after inhibition of miRNA-451a or miRNA-122-5p (Figures 4C–F). These findings suggest that inhibition of miR-451a and miR-122-5p may improve the function of circulating NK cells after acute ischemic stroke.

## Discussion

This study provides the first evidence that acute ischemic stroke alters the expression pattern of miRNA in human NK cells. As documented here, brain ischemia induces a significant loss of NK cell number and activity in the peripheral blood that is associated with infarct volume. MiRNA sequencing analysis revealed that miR-451a and miR-122-5p are dramatically upregulated in NK cells when they are suppressed after acute ischemic stroke. Notably, our *in vitro* results demonstrate the contribution of miR-451a and miR-122-5p to NK cell suppression after brain ischemia, suggesting a profound role of miRNA in stroke-induced impairment of NK cell-mediated immune defense.

The significantly altered expression pattern of miRNA in human NK cells supports the involvement of miRNA in stroke-induced loss of NK cell competence and impaired immune defense. Among the identified NK cell miRNAs that were altered after acute ischemic stroke, many are predicted to regulate a number of target genes and pathways associated with lymphocyte activation, mobilization, and host immune defense against pathogens. As a developing field, there is growing interest of miRNAs in ischemic stroke because of their potential biomarker and therapeutic applications (19–21). This study extends our understanding of miRNA expression and gene regulation in human stroke into NK cells as a specific immune cell subset that form the first innate defense against infections.

Coupling miRNA sequencing with RT-PCR verification, we identified miRNA-451a and miRNA-122-5p that may contribute to impaired NK cell-mediated immune defense after acute ischemic stroke. These findings provide clues to further clarify the immunosuppressive mechanism of human NK cells. Consistent with previous studies showing impaired NK cell response after stroke (4, 22), these findings suggest that miR-451a and miR-122-5p are possibly involved in the alteration in expression of NK cell surface markers CD69 and NKG2D, but whether miR-451a and miR-122-5p contribute to stroke-induced NK cell deficit needs to be further confirmed by NK cell functional assays.

There are also limitations in this study that should be addressed in future investigations. First, our results should be interpreted cautiously because of the relatively small sample size. Second, the clinical characteristics of our stroke and control subjects may not be representative of the general stroke population. Therefore, caveats should be taken when generalizing our results to other stroke patients. Third, although our results suggest the contribution of miR-451a and miR-122-5p to impaired NK cell competence after brain ischemia, we cannot exclude potential involvement of other miRNAs in such process, for example, miRNA-23a-3p has also been reported to inhibit NK cells function (23, 24). Lastly, although we predict that the inhibitory effects of miR-451a and miRNA-122-5p on NK cells are mediated by PI3K-AKT or cAMP signaling pathway based on our KEGG analysis and previous published literatures (15, 18, 25), future studies would be required to support this hypothesis.

In summary, this study provides novel evidence that brain ischemia significantly alters the expression pattern of human NK cell miRNAs. The altered miRNAs such as miR-451a and miR-122-5p may contribute to stroke-induced NK cell impairment and serve as biomarkers.

## Data Availability Statement

All datasets generated for this study are included in the article/Supplementary Materials.

## Ethics Statement

All experiments were approved by the Ethics Committee of Tianjin Medical University General Hospital. The patients/participants provided their written informed consent to participate in this study.

## Author Contributions

YK, SL, XC, HR, and BZ acquired and analyzed the data. ML, X-AZ, and HM interpreted the results. ML and X-AZ formulated the study concept, designed the study, and drafted the manuscript.

## Conflict of Interest

The authors declare that the research was conducted in the absence of any commercial or financial relationships that could be construed as a potential conflict of interest.
