# Supplementary material for: Brain Ischemia Significantly Alters microRNA Expression in Human Peripheral Blood Natural Killer Cells
Source: Front Immunol. 2020 May 14;11:759. doi: 10.3389/fimmu.2020.00759 (PMC7240012; doi:10.3389/fimmu.2020.00759)
Supplement: Supplementary file 1 [file Data_Sheet_1.docx]

**Supplementary data**

**Table 1. The characteristics of human subjects**


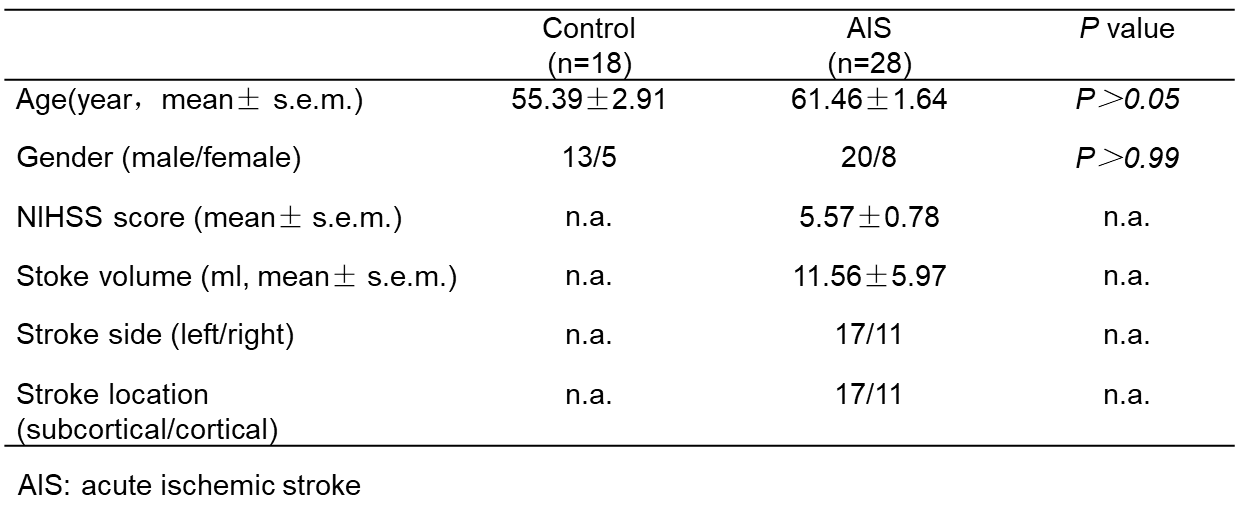

**Supplemental Figure 1. Verification of the tested miRNA expression using RT-PCR.**

RT-PCR analysis for the miR486-5p, miR23a-5p, miR92a-2-5p and miR4647 expression in NK cells from stroke patients and healthy control. n=8 per group. **p<0.01. Data are presented as means ± SEM.
